# *Streptococcus pneumoniae* Serotype-2 Childhood Meningitis in Bangladesh: A Newly Recognized Pneumococcal Infection Threat

**DOI:** 10.1371/journal.pone.0032134

**Published:** 2012-03-30

**Authors:** Samir K. Saha, Hassan M. Al Emran, Belal Hossain, Gary L. Darmstadt, Senjuti Saha, Maksuda Islam, Atique I. Chowdhury, Dona Foster, Aliya Naheed, Shams El Arifeen, Abdullah H. Baqui, Shamim A. Qazi, Stephen P. Luby, Robert F. Breiman, Mathuram Santosham, Robert E. Black, Derrick W. Crook, and Pneumococcal Study Group

**Affiliations:** 1 Department of Microbiology, Dhaka Shishu (Children) Hospital, Child Health Research Foundation, Dhaka, Bangladesh; 2 Bloomberg School of Public Health, Johns Hopkins University, Baltimore, Maryland, United States of America; 3 The Davidson Laboratory, Department of Molecular Genetics, University of Toronto, Toronto, Canada; 4 International Centre for Diarrhoeal Disease and Research, Dhaka, Bangladesh; 5 Infectious Diseases and Clinical Microbiology, University of Oxford, Oxford, United Kingdom; 6 Child and Adolescent Health Department, World Health Organization, Geneva, Switzerland; 7 Global Disease Detection and Emergency Response, Centers for Disease Control and Prevention, Atlanta, Georgia, United States of America; Centers for Disease Control & Prevention, United States of America

## Abstract

**Background:**

*Streptococcus pneumonia*e is a leading cause of meningitis in countries where pneumococcal conjugate vaccines (PCV) targeting commonly occurring serotypes are not routinely used. However, effectiveness of PCV would be jeopardized by emergence of invasive pneumococcal diseases (IPD) caused by serotypes which are not included in PCV. Systematic hospital based surveillance in Bangladesh was established and progressively improved to determine the pathogens causing childhood sepsis and meningitis. This also provided the foundation for determining the spectrum of serotypes causing IPD. This article reports an unprecedented upsurge of serotype 2, an uncommon pneumococcal serotype, without any known intervention.

**Methods and Findings:**

Cases with suspected IPD had blood or cerebrospinal fluid (CSF) collected from the beginning of 2001 till 2009. Pneumococcal serotypes were determined by capsular swelling of isolates or PCR of culture-negative CSF specimens. Multicenter national surveillance, expanded from 2004, identified 45,437 patients with suspected bacteremia who were blood cultured and 10,618 suspected meningitis cases who had a lumber puncture. Pneumococcus accounted for 230 culture positive cases of meningitis in children <5 years. Serotype-2 was the leading cause of pneumococcal meningitis, accounting for 20.4% (45/221; 95% CI 15%–26%) of cases. Ninety eight percent (45/46) of these serotype-2 strains were isolated from meningitis cases, yielding the highest serotype-specific odds ratio for meningitis (29.6; 95% CI 3.4–256.3). The serotype-2 strains had three closely related pulsed field gel electrophoresis types.

**Conclusions:**

*S. pneumoniae* serotype-2 was found to possess an unusually high potential for causing meningitis and was the leading serotype-specific cause of childhood meningitis in Bangladesh over the past decade. Persisting disease occurrence or progressive spread would represent a major potential infection threat since serotype-2 is not included in PCVs currently licensed or under development.

## Introduction

*Streptococcus pneumoniae* is estimated to cause >60,000 meningitis-associated deaths plus a poorly defined burden of long-term disability in children ≤5 years of age world-wide every year [1]. Meningitis is one of the most severe disease manifestations of a wide spectrum of invasive pneumococcal disease (IPD). Pneumonia and bacteremia account for the bulk of this invasive disease, representing an estimated 14.5 million cases and 735,000 deaths in HIV negative children globally per annum [2]. Pneumococcal conjugate vaccines (PCVs) offer the hope to prevent this burden of disease worldwide.

There are currently three PCVs licensed[PCV-7 (Prevnar 7®) and PCV-13 (Prevnar 13®)], respectively, manufactured by Pfizer and conjugated to diphtheria CRM197 protein; and PCV-10 (Synflorix®) a product of GlaxoSmithKline conjugated to Protein D, a non-typeable *Haemophilus influenzae* protein, as well as to tetanus toxoid and diphtheria toxoid proteins). Thus, a limited number of the >90 known pneumococcal capsular antigens that determine different serotypes, are currently incorporated in these vaccines. The capsular polysaccharide antigens included in these vaccines are based on the predominant serotypes causing IPD in a variety of settings around the world. This is not ideal for optimizing prevention of IPD in developing countries, since there is little cross-serotype protection and in the developing world and a substantial proportion of IPD is caused by non-vaccine serotypes [3]. Sudden rises in prevalence of currently rare serotypes would further undermine the effectiveness of these vaccines.

We preliminarily reported a high prevalence of serotype-2 IPD in Bangladesh [4], and here we now describe in detail *S. pneumoniae* serotype-2 as a leading cause of childhood pneumococcal meningitis based on 9 years (2001 to 2009) of surveillance.

## Methods

### Invasive pneumococcal surveillance in Bangladesh

Surveillance of bacteremia and meningitis etiology in children <5 years based at Dhaka Shishu Hospital (DSH) has been ongoing since January 2001. DSH is situated in Dhaka City (population 12 million) and is the only tertiary-care pediatric hospital in Bangladesh (population 150 million, the eighth most populous country in the world). Based on physician's judgment, blood culture was performed on suspected pneumonia and sepsis cases and lumbar puncture was undertaken on all cases of suspected meningitis. Through March 2004, data were recorded only for either blood or CSF cultures growing pneumococci.

Surveillance was extended to partial national pediatric surveillance of IPD on April 2004 by incorporating seven hospitals (DSH plus six new sites, one rural and five urban) in three districts [4], [5]. From 1 January 2009 onwards the surveillance was limited to the four high performing hospitals reporting the majority of cases. Pneumococcal isolates and any surplus CSF were sent to the microbiology laboratory at DSH. The age, gender, date of illness and residential addresses were collected on all cases. Locations of the case were collected by hand-held Magellan 350 Geographical Positioning System (GPS).

### Case definition of IPD

Pneumococcal meningitis was defined as isolation of *S. pneumoniae* from CSF, or from blood when the concurrent CSF was culture negative but contained ≥10 white cells ×10^6^/L. Additional cases of pneumococcal meningitis were ascertained by detection in CSF (with ≥10 white cells ×10^6^/L) of pneumococcal capsular antigens or pneumococcal-specific DNA sequence by PCR, see below. Cases of non-meningitis IPD consisted of those patients growing pneumococci from blood culture, who did not undergo lumbar puncture or on CSF examination had <10 white cells ×10^6^/L.

### Microbiological methods, detection of antimicrobial activity in CSF and non-culture detection of pneumococci in CSF

Blood and CSF were cultured as described previously [4]. Pneumococcal isolates were identified using standard methods [4], and preserved in media containing 2% skimmed milk, 3% tryptone, 10% glycerol and 0.5% glucose (STGG) at −70°C. Samples with surplus CSF were archived at −70°C. Pneumococcal isolates were serotyped by the conventional capsular swelling method as previously described [4]. Presence of antimicrobials in patients with ≥10 white cells ×10^6^/L was assayed on CSF using a disc-based agar plate bio-assay as previously described [4].

Pneumococcal antigen testing of surplus CSF was performed on all culture-negative CSF samples with ≥10 white cells ×10^6^/L, when blood cultures were also negative. The pneumococcal latex agglutination test (LAT) (Wellcogen Bacterial Antigen Kit; Remel Europe Ltd, Kent, UK) was initially used, and from 2004 onwards, if negative by LAT, CSF was next tested by immunochromatographic test (ICT) (Binax NOW *Streptococcus pneumoniae* test, Inverness Medical Professional Diagnostics, Princeton, NJ, USA), both according to the manufacturer's instructions.

PCR detection of pneumococcal sequences targeting the pneumolysin gene (*ply*-PCR) as previously described [6] was also performed on surplus CSF only for the specimens collected at DSH. PCR-based serotyping for the 38 most prevalent serotypes, using 38 pairs of primers as previously described [7], was performed on all non-culture pneumococcal positive specimens containing sufficient residual CSF.

### Genotyping of pneumococcal isolates

Isolates were analyzed using pulsed-field gel electrophoresis (PFGE) according to standard protocols [8], [9], [10]. Multi-locus sequence type (MLST) was performed and analyzed using eBURST as previously described [11], [12], [13].

### Ethics

This study on invasive pneumococcal disease surveillance was approved by the ethics review committee of the Bangladesh Institute of Child Health, Dhaka Shishu Hospital, and the International Centre for Diarrhoeal Disease Research, Bangladesh (ICDDR,B). Written informed consents were taken from parents or legal guardian of all participants for this surveillance.

### Data management and analysis

Data were entered into Epi-data and analyzed using STATA 9 (StataCorp, US). An empirical odds ratio (OR) for meningitis-causing potential was calculated for each serotype with ≥10 IPD cases relative to all the serotypes with <10 IPD cases, referred to here as ‘other serotypes’ (as the reference group), following the approach for calculating serotype-specific invasive ratios [14]. Serotype-specific ORs were calculated for each serotype causing culture-positive meningitis and total IPD using the following equation: OR = (*ad*)/(*bc*), where *a* is the number of meningitis-causing isolates for a specific serotype, *b* is the number of total IPD isolates for the same specific serotype, *c* is the number of meningitis-causing ‘other serotype’ isolates, and *d* is the number of total IPD ‘other serotype’ isolates. We interpret the odds ratio as a measure of serotype-specific propensity to cause meningitis.

### Role of the funding sources

The sponsors of the study had no role in study design, data collection, data analysis, data interpretation, or writing of the report. The corresponding author had full access to all the data in the study and had final responsibility for the decision to submit for publication.

## Results

### Culture-positive IPD cases

**Figure 1:**
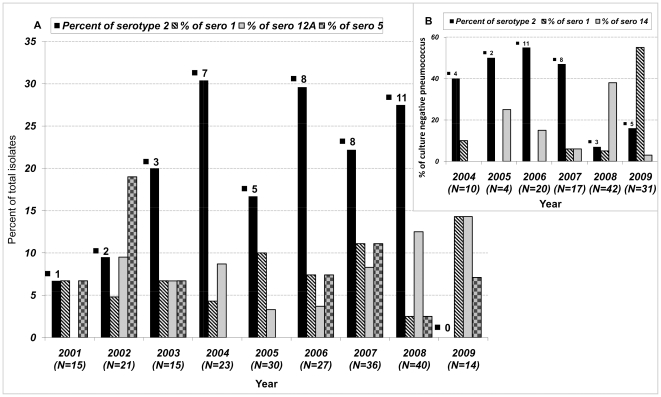
Cases of Pneumococcal Meningitis by Leading Serotypes Detected According to Culture or Non-Culture Methods. This depicts the percentage of meningitis cases <5 years by the leading serotypes identified by culture (A) and non-culture (B) detection methods. In brackets, (N), under the year along the x axis indicates the total number of serotype detected in that year. The number above each serotype-2 solid black bar in the histogram represents the number of isolates.

*S. pneumoniae* was isolated from 342 children <5 years old; 230 were cases of meningitis and 112 were cases of non-meningitis IPD. Of these isolates, 333 (221 meningitis and 112 non-meningitis) were serotyped; 6 were lost and 3 were non-typable and were excluded (Table 1 and Table S1, Table S2). Of the four leading serotypes, serotype-2 was the most common serotype causing meningitis during the observation period (n = 45; 20.4%; 95% CI 15%–26%) followed by serotype 12A (n = 17; 7.7%, CI 4%–11%), serotype 1 (n = 16; 7.2%, CI 4%–11%) and serotype 5 (n = 14; 6.3%, CI 3%–10%). For 6 of the 9 years of surveillance, 2003–2008, serotype 2 was the leading serotype causing meningitis, although only one case was isolated in 2001 and in 2009 no culture positive cases were identified (Figure 1).

**Table 1:** IPD Isolates from Children <5 years between 2001 and 2009.

	Isolates fully serotyped			Isolates excluded^d^			
Surveillance Category	Meningitis	Non-meningitis	Total	Missing^e^	Non-typable^f^	Total	Total all isolates
2001–2003^a^	51	6	57	3	2	5	62
2004–2008^b^	156	92	248	3	1	4	280
2009^C^	14	14	28	0	0	0	28
Total	221	112	333	6	3	9	342

a = Surveillance period which included only DSH.

b = Speriod which included DSH and 6 other hospitals.

c = Surveillance period which included DSH and 3 other hospitals.

d = Cases with these isolates were excluded from the analysis.

e = Missing indicates isolate was not retrievable from the isolate archive.

f = Non-typable indicates that the isolates were not among the known serotypes.

Of the 342 pneumococcal cases, 280 were recorded as part of the partial national pediatric surveillance commencing in 2004 for which denominator data was available. 45,437 patients were blood cultured and 10,618 suspected meningitis cases had lumbar puncture performed. Only 2,490(23%) of these yielded a cell count ≥10 white cells ×10^6^/L. Of these abnormal CSF specimens, 378(15%) grew a pathogen. Of these, 174 were *S. pneumoniae*, which was the leading species-specific cause of childhood meningitis, accounting for 46% (174/378; 95% CI 42%–50%) of cases that yielded a pathogen. Of the 45,473 potentially septic patients, 42,947 were considered non-meningitis cases, either on clinical grounds or because the CSF yielded <10 white cells ×10^6^/L. A pathogen was grown from 3,300 (7.7%) and of these, 106 (3.2%) were *S. pneumoniae*.

**Figure 2:**
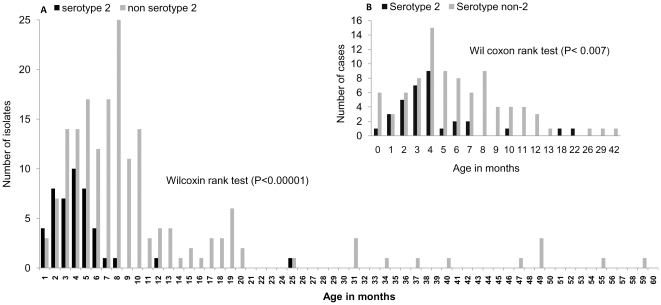
Age Distribution of Serotype-2 Pneumococcal Meningitis Cases Compared to All Other Serotypes. This depicts the frequency by age of the serotype-2 compared to non serotype 2 pneumococcal meningitis cases in children <5 years. The median and interquartile ranges were 3 months (1 to 4) and 7 months (4 to 9) respectively.

The median age for serotype-2 cases was 3 months compared to 7 months for other serotypes (P<0.00001; Wilcoxon rank test,). The age group difference between serotype-2 (median age 4 months) and other serotypes (median age 5 months) was similar for culture negative cases of pneumococcal meningitis; (P<0.007; Wilcoxon rank test) (Figure 2). Compared to other serotypes, the OR for serotype-2 causing meningitis (n = 45 isolates) versus total IPD (meningitis and bacteremia, n = 46 isolates) was 29.6 (95% CI: 3.4–256.3), p<0.001, the highest among all serotypes analyzed (Table 2).

**Table 2:** Meningitis-causing potential of serotype 2 compared to other serotypes.

Serotypes	Meningitis/total IPD (%)	Odd Ratio (95% CI¶)	p-value
2	45/46 (98%)	29.6 (3.4–256.3)	<0.001
1	16/35 (46%)	0.6 (0.3–1.2)	0.1
5	14/25 (56%)	0.8 (0.4–2.0)	0.7
12A	17/20 (85%)	3.7 (1.0–13.7)	0.03
14	11/17 (65%)	1.2 (0.4–3.5)	0.7
45	10/17 (59%)	0.9 (0.3–2.6)	0.9
6B	8/13 (62%)	1.1 (0.3–3.4)	0.9
7F	9/13 (69%)	1.5 (0.4–5.1)	0.5
18C	9/11 (82%)	3.0 (0.6–14.5)	0.2
Other¥	82/136 (60%)	1.§	

¶ CI =  confidence interval.

¥ 40 other serotypes; non-typable (N = 3) and missing strains (N = 6) were excluded from this analysis.

§ The reference category is: other serotypes (grouped).

### Ascertainment of further cases by non-culture methods and antimicrobial activity in CSF

Of the 2,112 culture-negative CSF samples from the partial national pediatric surveillance, 1,181 contained surplus CSF for further testing. These were processed by the LAT and ICT tests, of which 386 were positive for pneumococci, 84 were positive for *Haemophilus influenzae* type b and 22 for *Neisseria meningitidis*. Of the 687 samples negative by LAT and ICT tests, 17 were found to be positive by *ply*-PCR. Of the 403 LAT, ICT or *ply*-PCR positive CSF samples, 217 had sufficient amount for PCR serotyping; 124 were successfully typed, while 93 could not be typed.

Serotype-2, similar to culture positive cases, comprised the largest sub-set of the culture-negative cases, accounting for 33(27%; 95% CI 19%–34%) cases; 22 (18%, CI 11%–24%) were serotype-14, 21 were serotype-1 (17%, CI 10%–24%) and 48 consisted of 13 other serogroups/serotypes (Table S3). Fewer serotype-2 cases were detected in 2009, mirroring the decline observed in 2009 among culture positive cases (Figure 1).The large number of pneumococcal meningitis cases detected only by non-culture methods suggests that many had prior exposure to antibiotics. Sufficient CSF was available for antimicrobial substance testing from 639 CSF samples collected as part of the partial national pediatric hospital surveillance. Antimicrobial activity was detected in 348 (54%; CI 51%–58%) of these samples; 79%in the culture-negative group (n = 160) and 25% of the culture-positive group (n = 87) (Table S4).

### Geographic distribution of serotype-2 cases in Bangladesh

The geographical distribution of the cases was mapped to place of residence using GPS technology for cases identified during 2001 through 2009. Of the 78 serotype-2 meningitis cases (45 culture-positive and 33 detected by non-culture methods), 69 were traced. These were scattered widely throughout the surveillance area, with the highest concentration in Dhaka city, the main referral base for DSH and possessing the highest population density (Figure S1). Community health workers' visits to households and interviews with families of cases did not reveal any clustering within Dhaka city.

### Genotypic relatedness by PFGE and MLST

**Figure 3:**
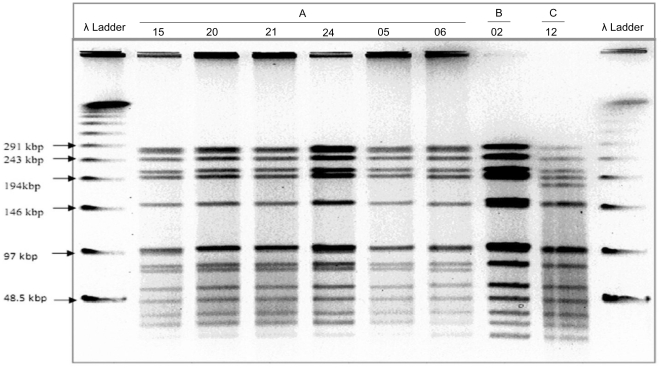
Restriction Fragments Generated by Pulsed-Field Gel Electrophoresis. Showing banding patterns of serotype 2 representing 3 different pulsetypes, A (N = 36), B (N = 4)& C (N = 1), detected from *Sma1* digested genomic DNA.

**Figure 4:**
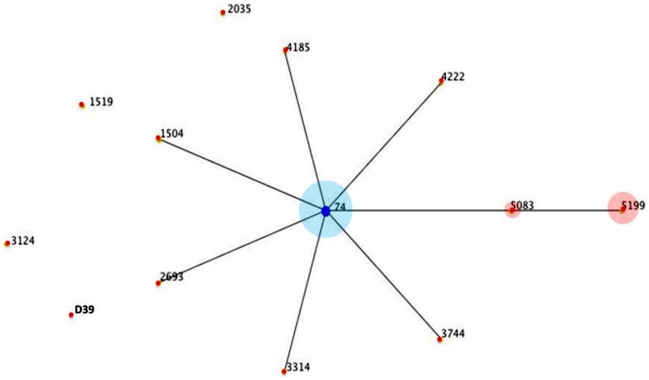
Depiction of eBURST analysis of the serotype 2 data available on http://www.mlst.net/ **.** This is a depiction of an eBURST analysis of the serotype 2 data available on http://www.mlst.net/. The founding sequence type (ST) of the only clonal complex is ST 74, marked by the dark blue node. For the Bangladeshi isolates, the majority (n = 23) belonged to ST 74. The two other sequence types 5083 (n = 3) and 5199 (n = 15) were single and double locus variants of sequence type 74 respectively. The relative proportions are reflected in the light blue and light pink circles around the respective nodes. Epidemiological data recorded on http://www.mlst.net indicates CC 74 is the dominant lineage recorded among those MLST and they originate mainly from West Africa.

Of the 46 serotype-2 isolates cultured, 41 were available for genotyping (Figure 3). PFGE identified three types (A, B & C) differing by only 2 or 3 bands and, therefore, would be regarded as closely related [9]. By MLST, these 41 isolates belonged to a single clonal complex, CC74, consisting of 3 sequence types (ST): ST 74 (n = 23) the ancestral type, a single locus variant ST 5083 (n = 3) and a double locus variant ST 5199 (n = 15). These latter two STs are unique to this study. This serotype-2 clonal complex is genetically unrelated to other rare serotype-2 sequence types on the http://www.mlst.net database; including the archetypal whole genome sequenced historic strain D39 designated ST 595(Figure 4 and Table S5).

## Discussion

This study shows that *S. pneumoniae* was the leading species-specific cause of childhood meningitis during the last decade in Bangladesh, accounting for 46% of culture-positive cases. Serotype-2 pneumococcus was the leading serotype-specific cause during the surveillance period, accounting for 20% of the culture positive meningitis cases with a very strong predilection for causing meningitis (OR = 29.6) compared with bacteremic IPD. Another distinctive feature of this pneumococcal serotype was that it affected younger children compared with all other serotypes and it also consisted of a genetic lineage of highly related STs.

Routine, comprehensive population-based surveillance is not established throughout Bangladesh [15], which prevents determination of the nation-wide incidence of meningitis. Consequently, the full extent and impact of serotype-2 pneumococcal meningitis is difficult to ascertain. The study, however, took advantage of rigorous, ongoing hospital-based surveillance with concomitant careful laboratory investigation of cases. Serotype-2 was also sporadically isolated during the period of passive surveillance from 1993 to 2000, when 15 cases were identified (∼ two cases per year) [16], [17]. Since the end of 2009, four culture-positive serotype-2 cases, all with meningitis, have been recorded (data not shown). Since we conducted surveillance in several large hospitals and used sophisticated laboratory methods to identify the serotypes, we are confident that serotype-2 pneumococcal meningitis over the observation period was the predominant cause of pneumococcal childhood meningitis. The recent observation of fewer cases may be chance variation or alternatively suggestive of epidemic like behavior typical of serotype-1, which is known to cause epidemics of IPD and specifically meningitis in West Africa [18], [19], [20].

The geographic range and burden of serotype-2 IPD in Bangladesh and surrounding countries is difficult to determine because active surveillance with serotype testing in regions surrounding Bangladesh is limited or non-existent. The number of cases identified in this study is likely to be an underestimate of the burden, as only a minority of sick children with symptoms of meningo-encephalitis present to hospitals in Bangladesh [21]. Also, the very substantial exposure to antibiotics (>50% of meningitis cases) likely reduced the detection of cases. For these reasons, it is possible that a major burden of serotype-2 pneumococcal meningitis could go unrecognized. Intriguingly, a small-scale report of 28 invasive pneumococcal disease patients from Nepal [22] included two cases (ranking 3^rd^ in serotype prevalence) caused by serotype-2, which indicates the existence of this serotype in a neighboring country. Furthermore, a wider spectrum of serotypes could contribute to pneumococcal meningitis, as 93 (43%) of the 217 antigen-positive CSF samples failed to yield a result on serotype-specific PCR that was limited to about a third of the 90 possible serotypes identified.

In the 1930 s, serotype-2 was a major cause of pneumococcal pneumonia but apparently not meningitis. In a large series of 991serotype-2 adult pneumonia cases observed in the US , 46% of which were bacteremic, only 24 cases (2.4%) manifested meningitis [23], [24]. Recent reports on 70 years of pneumococcal serotype secular trends from the USA and Denmark showed disappearance in the 1960 s of serotype-2 from reports of IPD [25], [26]. In the 1970 s, serotype-2 was still a major cause of pneumonia among South African miners [27], but was not overtly described as having a propensity for causing meningitis. Serotype-2 IPD is now rare in South Africa [28], [29]. No recent reports of high-incidence serotype-2 IPD could be found in the published literature.

It is not possible to determine whether Bangladesh is a major persisting reservoir of serotype-2 pneumococci or whether this serotype-2 meningitis-causing lineage is emerging as a new variant. Genetically, the lineage occurring in Bangladesh is distinct from the historic strain D39 isolated in the early 1900 s.The meningitis-causing feature of the serotype-2 pneumococcal lineage reported here is unusual or even unparalleled for a pneumococcus in having such a high odds for causing meningitis. In studies of meningitis from Africa, compared to serotype-1, serotype 2 did not account for a prominent proportion of meningitis cases [18], [19], [20]. It raises the question of whether a variant with specific central nervous system tropism has recently evolved within the clonal complex CC74 in Bangladesh which differentiates it from the members of the same lineage observed in West Africa [11]. Typically, such a new bacterial variant would undergo clonal expansion yielding indistinguishable isolates. The PGFE and MLST typing results are broadly consistent with such a phenomenon. However, the MLST data indicate there are three closely related STs: ST 74, ST 5083 and ST 5199 (the latter two being unique to Bangladesh) all belonging to the same clonal complex CC 74. These data are commensurate with these three members of CC74 having circulated for sufficient time to accumulate such sequence diversity. The three STs occurred consistently over the observation period, suggesting that evolution of this diversity antedated the sampling period. A better understanding of the possible genetic basis for this meningitis-causing trait awaits further investigation, including whole genomic sequence analysis and comparative genomics, which is ongoing.

The high proportion of meningitis associated with this serotype-2 pneumococcus strain highlights the potential for a serotype that was perceived to have nearly vanished to materialize as a major cause of IPD and pneumococcal meningitis in particular. Owing to its rarity in recent reports of IPD, serotype-2 has not been deemed a likely candidate for inclusion in any currently planned conjugate vaccine. It is unclear whether serotype-2 represented by this clonal complex will remain a dominant cause of meningitis in Bangladesh, spread to neighboring countries or even re-emerge as a major cause of IPD in regions where it had disappeared.

This study confirms the importance of ongoing surveillance before and after vaccine introduction, as well as the potential for substantial shifts in serotype profiles in the absence of vaccine introduction. It also indicates the need for alternate vaccine candidates that provide protection across serotypes such as protein-based vaccine candidates.

## Supporting Information

Figure S1 **The Geographical Distribution of the Pneumococcal Serotype-2 Cases.**
(TIFF)

Table S1 **Year of pneumococcal isolation from DSH and network of 6 hospitals.**
(DOCX)

Table S2 **Serotype of all IPD isolates from culture positive cases <5 years.**
(DOCX)

Table S3 **Serotype of non-culture CSF results.**
(DOCX)

Table S4 **Antibiotic exposure in cases of meningitis.**
(DOCX)

Table S5 **Genotypes of 41 serotype 2 isolates by PFGE and MLST.**
(DOCX)
