# Supplementary figures and images for: Streptococcus pneumoniae Serotype-2 Childhood Meningitis in Bangladesh: A Newly Recognized Pneumococcal Infection Threat
Source: PLoS One. 2012 Mar 30;7(3):e32134. doi: 10.1371/journal.pone.0032134 (PMC3316528; doi:10.1371/journal.pone.0032134)

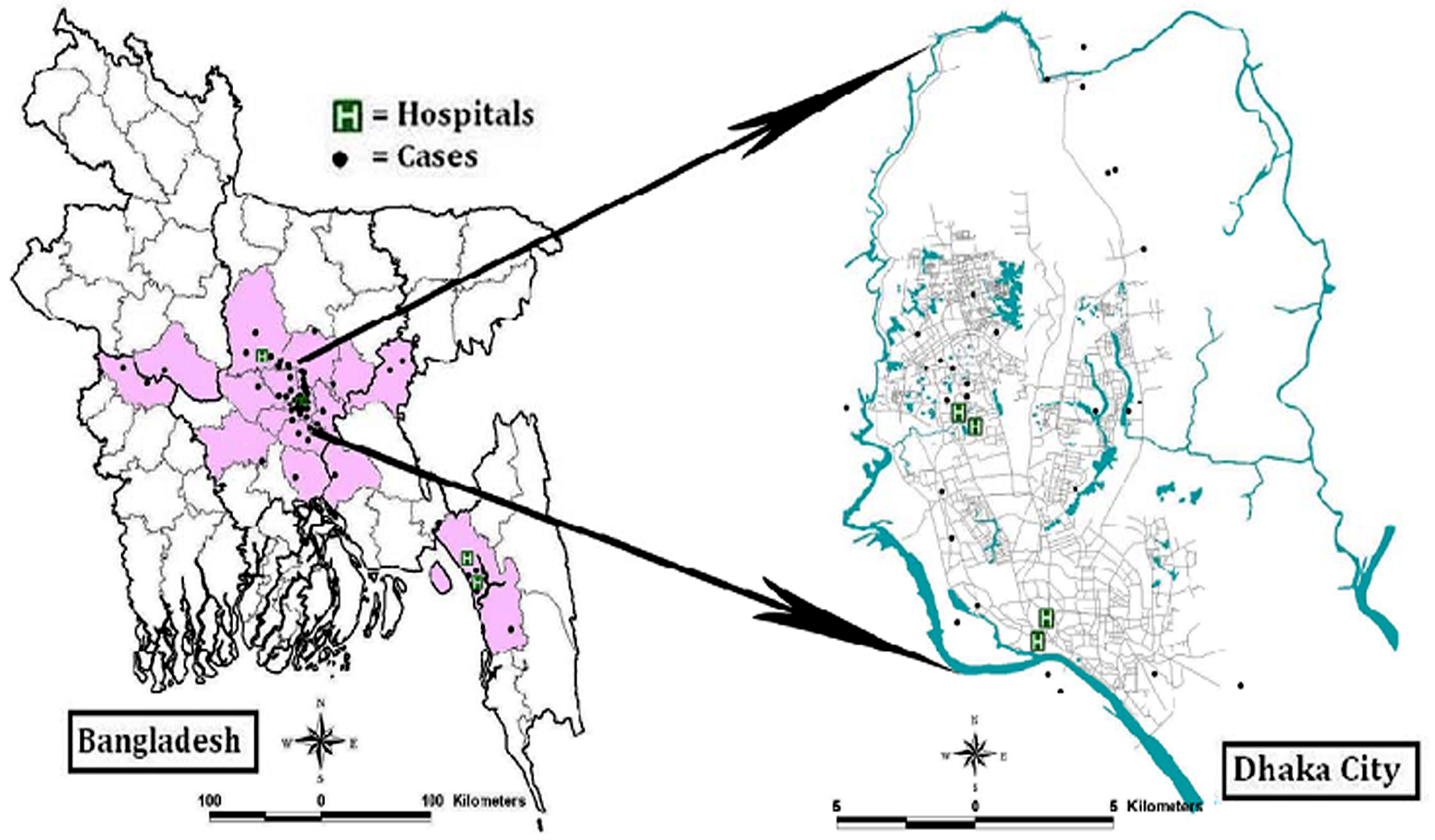

Supplement: Figure S1 — The Geographical Distribution of the Pneumococcal Serotype-2 Cases. (TIFF) [file pone.0032134.s001.tif]
