# Supplementary material for: Streptococcus pneumoniae Serotype-2 Childhood Meningitis in Bangladesh: A Newly Recognized Pneumococcal Infection Threat
Source: PLoS One. 2012 Mar 30;7(3):e32134. doi: 10.1371/journal.pone.0032134 (PMC3316528; doi:10.1371/journal.pone.0032134)
Supplement: Table S1 — Year of pneumococcal isolation from DSH and network of 6 hospitals. (DOCX) [file pone.0032134.s002.docx]

**Table S1. Year of pneumococcal isolation from DSH and network of 6 hospitals**

| **Manifestion** | **Hospital Serotype** | | **Year of Isolation** | | | | | | | | |  |
| --- | --- | --- | --- | --- | --- | --- | --- | --- | --- | --- | --- | --- |
|  |  |  | **2001** | **2002** | **2003** | **2004** | **2005** | **2006** | **2007** | **2008** | **2009** | **Total** |
| Meningitis | Dhaka Shishu Hospital (DSH) | 1 | 1 | 1 | 1 | 1 | 1 | 0 | 1 | 0 | 1 | 7 |
|  |  | 2 | 1 | 2 | 3 | 6 | 5 | 5 | 3 | 4 | 0 | 29 |
|  |  | 5 | 1 | 4 | 1 | 0 | 0 | 1 | 0 | 1 | 1 | 9 |
|  |  | 6A+6B | 1 | 1 | 2 | 0 | 4 | 0 | 1 | 0 | 0 | 9 |
|  |  | 7F | 0 | 1 | 0 | 0 | 0 | 0 | 0 | 0 | 1 | 2 |
|  |  | 10F+10A | 1 | 0 | 0 | 2 | 0 | 0 | 0 | 0 | 3 | 6 |
|  |  | 12A+12F | 0 | 2 | 1 | 0 | 1 | 1 | 1 | 2 | 3 | 11 |
|  |  | 14 | 0 | 1 | 1 | 1 | 0 | 0 | 2 | 0 | 0 | 5 |
|  |  | 18 | 0 | 2 | 0 | 1 | 1 | 2 | 3 | 2 | 1 | 12 |
|  |  | 19A+19F | 1 | 1 | 0 | 1 | 0 | 1 | 0 | 1 | 0 | 5 |
|  |  | 23F | 2 | 0 | 0 | 0 | 0 | 0 | 1 | 0 | 0 | 3 |
|  |  | 38 | 0 | 0 | 0 | 0 | 0 | 0 | 1 | 1 | 0 | 2 |
|  |  | 45 | 1 | 2 | 0 | 1 | 0 | 0 | 1 | 1 | 0 | 6 |
|  |  | Other | 6 | 4 | 6 | 1 | 2 | 2 | 1 | 3 | 1 | 26 |
|  |  | Total | 15 | 21 | 15 | 14 | 14 | 12 | 15 | 15 | 11 | 132 |
|  | 6 other hospitals in the network | 1 |  |  |  | 0 | 2 | 2 | 3 | 1 | 1 | 9 |
|  |  | 2 |  |  |  | 1 | 0 | 3 | 5 | 7 | 0 | 16 |
|  |  | 5 |  |  |  | 0 | 0 | 1 | 4 | 0 | 0 | 5 |
|  |  | 6A+6B |  |  |  | 1 | 1 | 0 | 0 | 1 | 2 | 5 |
|  |  | 7F |  |  |  | 0 | 4 | 1 | 2 | 0 | 0 | 7 |
|  |  | 10F+10A |  |  |  | 0 | 0 | 1 | 0 | 0 | 0 | 1 |
|  |  | 12A+12F |  |  |  | 2 | 0 | 1 | 2 | 3 | 0 | 8 |
|  |  | 14 |  |  |  | 1 | 2 | 0 | 1 | 2 | 0 | 6 |
|  |  | 18 |  |  |  | 0 | 2 | 2 | 1 | 4 | 0 | 9 |
|  |  | 19A+19F |  |  |  | 0 | 0 | 0 | 0 | 2 | 0 | 2 |
|  |  | 23F |  |  |  | 0 | 1 | 0 | 0 | 1 | 0 | 2 |
|  |  | 45 |  |  |  | 2 | 0 | 0 | 2 | 0 | 0 | 4 |
|  |  | Other |  |  |  | 2 | 4 | 4 | 1 | 4 | 0 | 15 |
|  |  | Total |  |  |  | 9 | 16 | 15 | 21 | 25 | 3 | 89 |
| Non-meningitis | Dhaka Shishu Hospital (DSH) | 1 | 0 | 0 | 0 | 2 | 2 | 1 | 0 | 1 | 2 | 8 |
|  |  | 5 | 0 | 1 | 0 | 1 | 0 | 0 | 0 | 0 | 0 | 2 |
|  |  | 6A+6B | 0 | 0 | 0 | 0 | 0 | 0 | 0 | 1 | 2 | 3 |
|  |  | 7F | 0 | 1 | 0 | 0 | 0 | 0 | 0 | 0 | 0 | 1 |
|  |  | 10F+10A | 0 | 0 | 0 | 0 | 0 | 0 | 0 | 1 | 0 | 1 |
|  |  | 12A+12F | 0 | 0 | 0 | 0 | 0 | 0 | 0 | 1 | 1 | 2 |
|  |  | 18 | 0 | 0 | 0 | 0 | 0 | 0 | 0 | 2 | 0 | 2 |
|  |  | 19A+19F | 0 | 2 | 0 | 0 | 0 | 2 | 1 | 1 | 1 | 7 |
|  |  | 23F | 0 | 1 | 0 | 0 | 0 | 0 | 0 | 1 | 1 | 3 |
|  |  | 45 | 0 | 0 | 0 | 0 | 1 | 1 | 0 | 0 | 0 | 2 |
|  |  | Other | 0 | 1 | 0 | 1 | 2 | 1 | 1 | 0 | 1 | 7 |
|  |  | Total | 0 | 6 | 0 | 4 | 5 | 5 | 2 | 8 | 8 | 38 |
|  | 6 other hospitals in the network | 1 |  |  |  | 0 | 1 | 4 | 3 | 2 | 1 | 11 |
|  |  | 2 |  |  |  | 0 | 0 | 0 | 0 | 1 | 0 | 1 |
|  |  | 5 |  |  |  | 0 | 3 | 3 | 1 | 1 | 1 | 9 |
|  |  | 6A+6B |  |  |  | 0 | 0 | 0 | 2 | 1 | 2 | 5 |
|  |  | 7F |  |  |  | 0 | 0 | 1 | 1 | 0 | 1 | 3 |
|  |  | 10F+10A |  |  |  | 0 | 0 | 0 | 1 | 0 | 0 | 1 |
|  |  | 12A+12F |  |  |  | 0 | 0 | 2 | 0 | 0 | 0 | 2 |
|  |  | 14 |  |  |  | 0 | 4 | 1 | 1 | 0 | 0 | 6 |
|  |  | 18 |  |  |  | 0 | 1 | 1 | 1 | 1 | 0 | 4 |
|  |  | 19A+19F |  |  |  | 0 | 3 | 1 | 1 | 2 | 1 | 8 |
|  |  | 23F |  |  |  | 1 | 1 | 0 | 0 | 0 | 0 | 2 |
|  |  | 38 |  |  |  | 0 | 1 | 1 | 0 | 1 | 0 | 3 |
|  |  | 45 |  |  |  | 0 | 1 | 1 | 3 | 0 | 0 | 5 |
|  |  | Other |  |  |  | 0 | 1 | 3 | 4 | 6 | 0 | 14 |
|  |  | Total |  |  |  | 1 | 16 | 18 | 18 | 15 | 6 | 74 |
