# Supplementary material for: Streptococcus pneumoniae Serotype-2 Childhood Meningitis in Bangladesh: A Newly Recognized Pneumococcal Infection Threat
Source: PLoS One. 2012 Mar 30;7(3):e32134. doi: 10.1371/journal.pone.0032134 (PMC3316528; doi:10.1371/journal.pone.0032134)
Supplement: Table S2 — Serotype of all IPD isolates from culture positive cases <5 years. (DOCX) [file pone.0032134.s003.docx]

**Table S2. Serotype of all IPD isolates from culture positive cases < 5 years.**

|  | **Meningitis** | | **Non-meningitis** | |
| --- | --- | --- | --- | --- |
| **Serotype** | **N** | **%** | **N** | **%** |
| 2 | 45 | 20.40% | 1* | 0.90% |
| 12A | 17 | 7.70% | 3 | 2.70% |
| 1 | 16 | 7.20% | 19 | 17.00% |
| 5 | 14 | 6.30% | 11 | 9.80% |
| 14 | 11 | 5.00% | 6 | 5.40% |
| 45 | 10 | 4.50% | 7 | 6.30% |
| 18C | 9 | 4.10% | 2 | 1.80% |
| 7F | 9 | 4.10% | 4 | 3.60% |
| 6B | 8 | 3.60% | 5 | 4.50% |
| 18F | 7 | 3.20% | 0 | 0.00% |
| 6A | 6 | 2.70% | 3 | 2.70% |
| 18A | 5 | 2.30% | 3 | 2.70% |
| 19F | 5 | 2.30% | 3 | 2.70% |
| 23F | 5 | 2.30% | 5 | 4.50% |
| 8 | 5 | 2.30% | 1 | 0.90% |
| 10F | 4 | 1.80% | 2 | 1.80% |
| 21 | 4 | 1.80% | 0 | 0.00% |
| 10A | 3 | 1.40% | 0 | 0.00% |
| 20 | 3 | 1.40% | 1 | 0.90% |
| 29 | 3 | 1.40% | 0 | 0.00% |
| 4 | 3 | 1.40% | 0 | 0.00% |
| 12F | 2 | 0.90% | 1 | 0.90% |
| 19A | 2 | 0.90% | 12 | 10.70% |
| 24 | 2 | 0.90% | 1 | 0.90% |
| 33 | 2 | 0.90% | 0 | 0.00% |
| 33B | 2 | 0.90% | 0 | 0.00% |
| 33F | 2 | 0.90% | 1 | 0.90% |
| 38 | 2 | 0.90% | 3 | 2.70% |
| 48 | 2 | 0.90% | 0 | 0.00% |
| 9V | 2 | 0.90% | 1 | 0.90% |
| 16F | 1 | 0.50% | 1 | 0.90% |
| 23B | 1 | 0.50% | 0 | 0.00% |
| 25 | 1 | 0.50% | 0 | 0.00% |
| 27 | 1 | 0.50% | 0 | 0.00% |
| 28A | 1 | 0.50% | 0 | 0.00% |
| 34 | 1 | 0.50% | 0 | 0.00% |
| 35A | 1 | 0.50% | 0 | 0.00% |
| 35F | 1 | 0.50% | 1 | 0.90% |
| 39 | 1 | 0.50% | 0 | 0.00% |
| 40 | 1 | 0.50% | 0 | 0.00% |
| 7C | 1 | 0.50% | 0 | 0.00% |
| 11A | 0 | 0.00% | 1 | 0.90% |
| 15 | 0 | 0.00% | 1 | 0.90% |
| 15A | 0 | 0.00% | 2 | 1.80% |
| 15B | 0 | 0.00% | 1 | 0.90% |
| 15F | 0 | 0.00% | 1 | 0.90% |
| 18B | 0 | 0.00% | 1 | 0.90% |
| 22F | 0 | 0.00% | 1 | 0.90% |
| 23A | 0 | 0.00% | 2 | 1.80% |
| 25F | 0 | 0.00% | 1 | 0.90% |
| 3 | 0 | 0.00% | 2 | 1.80% |
| 35B | 0 | 0.00% | 2 | 1.80% |
| Total serotype detected | 221 | 100% | 112 | 100% |
| Non-typable | 3 |  | 0 |  |
| Missing | 6 |  | 0 |  |
| Total | 230 |  | 112 |  |

*This case was admitted in status epilepticus and died without a

lumber puncture
