# Supplementary material for: Streptococcus pneumoniae Serotype-2 Childhood Meningitis in Bangladesh: A Newly Recognized Pneumococcal Infection Threat
Source: PLoS One. 2012 Mar 30;7(3):e32134. doi: 10.1371/journal.pone.0032134 (PMC3316528; doi:10.1371/journal.pone.0032134)
Supplement: Table S3 — Serotype of non-culture CSF results. (DOCX) [file pone.0032134.s004.docx]

**Table S3. Serotype of non-culture CSF results**

| **Serotype detected by multiplex PCR** | **Frequency of children <5 years** | **Percent of children <5 years** |
| --- | --- | --- |
| 1 | 21 | 17% |
| 10F | 1 | 1% |
| 12 | 4 | 3% |
| 14 | 22 | 18% |
| 18 | 5 | 4% |
| 2 | 33 | 27% |
| 23F | 1 | 1% |
| 24 | 6 | 5% |
| 34 | 1 | 1% |
| 38 | 3 | 2% |
| 4 | 2 | 2% |
| 45 | 3 | 2% |
| 5 | 5 | 4% |
| 6 | 9 | 7% |
| 7F | 5 | 4% |
| 8 | 3 | 2% |
| Total serotype detected | 124 | 100% |
| Non-typable using available primers | 93 |  |
| Insufficient sample for testing | 186 |  |
| Total culture negative pneumococcal cases | 403 |  |
