# Supplementary material for: Streptococcus pneumoniae Serotype-2 Childhood Meningitis in Bangladesh: A Newly Recognized Pneumococcal Infection Threat
Source: PLoS One. 2012 Mar 30;7(3):e32134. doi: 10.1371/journal.pone.0032134 (PMC3316528; doi:10.1371/journal.pone.0032134)
Supplement: Table S4 — Antibiotic exposure in cases of meningitis. (DOCX) [file pone.0032134.s005.docx]

**Table S4.** **Antibiotic exposure in cases of meningitis**

|  | **Antibiotic assay** |
| --- | --- |
| **Pneumococcal detection** | **Positive/total (%; 95% CI)** |
| Culture positive | 22/87 (25; 16-34)* |
| Antigen/PCR positive | 127/160 (79; 73-86)* |
| Negative | 199/392 (51; 46-56) |
| Total | 348/639 (54; 51-58) |

* χ^2^_1_ = 68; P < 0.0001
