# Supplementary material for: Streptococcus pneumoniae Serotype-2 Childhood Meningitis in Bangladesh: A Newly Recognized Pneumococcal Infection Threat
Source: PLoS One. 2012 Mar 30;7(3):e32134. doi: 10.1371/journal.pone.0032134 (PMC3316528; doi:10.1371/journal.pone.0032134)
Supplement: Table S5 — Genotypes of 41 serotype 2 isolates by PFGE and MLST. (DOCX) [file pone.0032134.s006.docx]

**Table S5. Genotypes of 41 serotype 2 isolates by PFGE and MLST**

| **Sl. No.** | **Age in month** | **Year** | **aroE*** | **gdh*** | **gki*** | **recP*** | **spi*** | **xpt*** | **ddl*** | **ST^§^** | **PFGE type** |
| --- | --- | --- | --- | --- | --- | --- | --- | --- | --- | --- | --- |
| 1 | 5 | 2003 | 2 | 13 | 4 | 5 | 234 | 6 | 14 | 5199 | B |
| 2 | 7 | 2003 | 2 | 13 | 4 | 5 | 234 | 6 | 14 | 5199 | B |
| 3 | 2 | 2004 | 2 | 13 | 4 | 5 | 234 | 6 | 14 | 5199 | A |
| 4 | 4 | 2007 | 2 | 13 | 4 | 1 | 6 | 6 | 14 | 74 | A |
| 5 | 1 | 2007 | 2 | 13 | 4 | 1 | 6 | 6 | 14 | 74 | A |
| 6 | 0 | 2008 | 2 | 13 | 4 | 5 | 234 | 6 | 14 | 5199 | A |
| 7 | 1 | 2008 | 2 | 13 | 4 | 5 | 234 | 6 | 14 | 5199 | A |
| 8 | 4 | 2004 | 2 | 13 | 4 | 1 | 6 | 6 | 14 | 74 | A |
| 9 | 3 | 2004 | 2 | 13 | 4 | 5 | 234 | 6 | 14 | 5199 | A |
| 10 | 5 | 2004 | 2 | 13 | 4 | 1 | 6 | 6 | 14 | 74 | A |
| 11 | 2 | 2004 | 2 | 13 | 4 | 1 | 6 | 6 | 14 | 74 | A |
| 12 | 1 | 2004 | 2 | 13 | 4 | 5 | 234 | 6 | 14 | 5199 | A |
| 13 | 4 | 2005 | 2 | 13 | 4 | 5 | 234 | 6 | 14 | 5199 | A |
| 14 | 11 | 2005 | 2 | 13 | 4 | 1 | 6 | 6 | 14 | 74 | A |
| 15 | 3 | 2005 | 2 | 13 | 4 | 5 | 234 | 6 | 14 | 5199 | A |
| 16 | 3 | 2005 | 2 | 13 | 4 | 1 | 6 | 6 | 14 | 74 | A |
| 17 | 2 | 2005 | 2 | 13 | 4 | 5 | 234 | 6 | 14 | 5199 | C |
| 18 | 3 | 2006 | 2 | 13 | 4 | 5 | 6 | 6 | 14 | 5083 | A |
| 19 | 5 | 2006 | 2 | 13 | 4 | 1 | 6 | 6 | 14 | 74 | A |
| 20 | 3 | 2006 | 2 | 13 | 4 | 1 | 6 | 6 | 14 | 74 | A |
| 21 | 3 | 2006 | 2 | 13 | 4 | 1 | 6 | 6 | 14 | 74 | A |
| 22 | 1 | 2006 | 2 | 13 | 4 | 1 | 6 | 6 | 14 | 74 | A |
| 23 | 1 | 2007 | 2 | 13 | 4 | 1 | 6 | 6 | 14 | 74 | A |
| 24 | 1 | 2007 | 2 | 13 | 4 | 1 | 6 | 6 | 14 | 74 | A |
| 25 | 4 | 2008 | 2 | 13 | 4 | 1 | 6 | 6 | 14 | 74 | A |
| 26 | 0 | 2008 | 2 | 13 | 4 | 1 | 6 | 6 | 14 | 74 | A |
| 27 | 5 | 2008 | 2 | 13 | 4 | 1 | 6 | 6 | 14 | 74 | A |
| 28 | 3 | 2006 | 2 | 13 | 4 | 1 | 6 | 6 | 14 | 74 | A |
| 29 | 1 | 2007 | 2 | 13 | 4 | 1 | 6 | 6 | 14 | 74 | A |
| 30 | 4 | 2007 | 2 | 13 | 4 | 1 | 6 | 6 | 14 | 74 | A |
| 31 | 4 | 2008 | 2 | 13 | 4 | 5 | 234 | 6 | 14 | 5199 | B |
| 32 | 1 | 2008 | 2 | 13 | 4 | 5 | 6 | 6 | 14 | 5083 | A |
| 33 | 24 | 2004 | 2 | 13 | 4 | 5 | 234 | 6 | 14 | 5199 | A |
| 34 | 0 | 2007 | 2 | 13 | 4 | 1 | 6 | 6 | 14 | 74 | A |
| 35 | 3 | 2008 | 2 | 13 | 4 | 5 | 234 | 6 | 14 | 5199 | A |
| 36 | 4 | 2008 | 2 | 13 | 4 | 5 | 234 | 6 | 14 | 5199 | A |
| 37 | 2 | 2006 | 2 | 13 | 4 | 1 | 6 | 6 | 14 | 74 | A |
| 38 | 6 | 2007 | 2 | 13 | 4 | 5 | 234 | 6 | 14 | 5199 | A |
| 39 | 0 | 2008 | 2 | 13 | 4 | 5 | 6 | 6 | 14 | 5083 | A |
| 40 | 3 | 2008 | 2 | 13 | 4 | 1 | 6 | 6 | 14 | 74 | B |
| 41 | 3 | 2008 | 2 | 13 | 4 | 1 | 6 | 6 | 14 | 74 | A |

* aroE, gdh, gki, recP, spi, xpt and ddl are the genes included in *S. pneumoniae* MLST.

^§^ ST = MLST sequence type
